# Oxytocin levels and self-reported anxiety during interactions between humans and cows

**DOI:** 10.3389/fpsyg.2023.1252463

**Published:** 2023-09-14

**Authors:** Bente Berget, Judit Vas, Gunn Pedersen, Kerstin Uvnäs-Moberg, Ruth C. Newberry

**Affiliations:** ^1^Faculty of Health and Social Sciences, Department of Health, Social and Welfare Studies, University of South-Eastern Norway, Borre, Norway; ^2^Department of Health and Society, NORCE Norwegian Research Centre AS, Kristiansand, Norway; ^3^Faculty of Biosciences, Department of Animal and Aquacultural Sciences, Norwegian University of Life Sciences, Ås, Norway; ^4^Department of Animal Environment and Health, Section of Anthrozoology and Applied Ethology, Swedish University of Agricultural Sciences, Skara, Sweden

**Keywords:** animal-assisted intervention, cow, human–animal interaction, oxytocin, anxiety, green care

## Abstract

**Introduction:**

Positive social interactions with farm animals may have therapeutic benefits on humans by increasing brain oxytocin secretion, as inferred from circulating oxytocin levels. The aim of this observational study was to investigate acute changes in human plasma oxytocin levels and state anxiety associated with interactions with dairy cows.

**Methods:**

Data were collected from 18 healthy female nursing students who performed stroking and brushing of an unfamiliar cow for 15 min. Blood samples were drawn before entering the cowshed (T1, baseline), and after 5 (T2) and 15 (T3) min of interaction with a cow. At T1 and T3, the students filled out the Norwegian version of the Spielberger State-Trait Anxiety Inventory-State Subscale (STAI-SS).

**Results:**

Across participants, no significant changes in average plasma oxytocin concentration were detected between time points (p>0.05). There was, however, a modest decline in the STAI-SS scores between T1 and T3 (p=0.015) and a positive correlation between the change in individual level of state anxiety between T1 and T3 and the change in OT concentration of the same individual between T2 and T3 (p = 0.045).

**Discussion:**

The results suggest that friendly social interactions with cows are beneficial in lowering state anxiety, but any relationship with release of OT into the circulation was complex and variable across individuals. The acute reduction in state anxiety lends support to the value of interacting with farm animals in the context of Green Care for people with mental health challenges.

## 1. Introduction

Being in the same room or interacting with a companion animal in an experimental setting or an animal-assisted intervention (AAI) can reduce cardiovascular and endocrinological stress parameters (reviewed in Friedmann et al., 2015). For example, Friedmann et al. (1983) found that the presence of a dog lowered blood pressure in children while they were reading or resting compared to when no dog was present. Multiple other studies (e.g., Grossberg and Alf, 1985; Vormbrock and Grossberg, 1988; Allen et al., 1991; Nagengast et al., 1997; Allen et al., 2002; Handlin et al., 2018) have reported changes in heart rate, heart rate variability, and blood pressure indicating a more relaxed state after interacting with companion animals, particularly after stroking them. Endocrine markers such as cortisol levels can also be influenced by companion animal contact (e.g., Barker et al., 2005; Cole et al., 2007; Viau et al., 2010). Furthermore, friendly interactions with animals are reported to reduce anxiety (Shiloh et al., 2003; Cole et al., 2007) and depression (Souter and Miller, 2007). A stronger anxiolytic effect of human-animal interactions has been found when there is a bond between the partners (e.g., own dog vs. unfamiliar dog: Odendaal and Meintjes, 2003; Handlin et al., 2011). However, findings from AAI with dogs indicate that a personal bond is not essential (e.g., Friedmann et al., 1983).

Oxytocin (OT) may play a central role in the anxiolytic effect of social interactions (Powell et al., 2019). Oxytocin is a neuropeptide produced centrally in the paraventricular nucleus (PVN) and supraoptic nucleus of the hypothalamus and released into the circulation via the posterior pituitary. Oxytocin is both concurrently and independently secreted within the brain, where it influences behavioral and emotional responses. In particular, parvocellular neurons of the PVN project to multiple brain regions involved in stress regulation including the amygdala, locus coeruleus, median eminence, and areas involved in autonomic nervous control (Uvnäs-Moberg et al., 2011). Oxytocin is also expressed in peripheral tissues including cardiovascular tissue (Jankowski et al., 2000). Due to the blood–brain barrier, circulating OT does not freely enter the brain and intracerebral OT release does not readily contribute directly to the circulating level of OT (Ermisch et al., 1973). However, circular nonapeptide OT is metabolized into OT fragments, some of which have been linked to touch-induced anti-stress effects under autonomic nervous control (Uvnäs Moberg et al., 2019). Oxytocin fragments may pass through the blood–brain barrier into the circulation and be detected in plasma by immunoassay along with whole OT (Jones et al., 1983). For these reasons, measurement of peripheral OT levels may contribute to the understanding of OT-mediated central effects.

Oxytocin release can be stimulated by social interaction and has been associated with prosocial behavior in mammalian species including humans (Heinrichs et al., 2009; but see Leng et al., 2022). Oxytocin may also play a role in interactions between humans and other animals (Uvnäs-Moberg, 1998; Beetz et al., 2012; Powell et al., 2019). Rapid increases in blood, urine or salivary OT have been reported in humans when interacting with their dog (e.g., Odendaal and Meintjes, 2003; Handlin et al., 2011, 2012), especially in women (Miller et al., 2009). To a lesser extent, interaction with a friendly unfamiliar dog has been reported to raise plasma OT, with the increase being detected 5 to 24 min after commencing an interaction and coinciding with a reduction in blood pressure (Odendaal, 2000). Additionally, elevated peripheral OT has been found in dogs when interacting with humans (e.g., Odendaal and Meintjes, 2003; Handlin et al., 2011, 2012; Rehn et al., 2014; López-Arjona et al., 2021), and intranasal OT administration to dogs has been reported to stimulate positive social interactions with people (Romero et al., 2014; Hernádi et al., 2015). In female cat owners, a rise in peripheral OT concentration during a 15-min interaction was correlated with gentle petting, hugging/kissing and skin contact with the cat (Johnson et al., 2021). A variety of non-noxious sensory stimuli associated with social contact, such as gentle touch, eye contact, verbal contact, and familiar odors, have been associated with a rise in peripheral OT (Uvnäs-Moberg, 1998; Nagasawa et al., 2009; Seltzer et al., 2010), including pleasurable gentle touch on the forearms with a brush (Portnova et al., 2020).

Although most of the available studies on AAI concern interaction with dogs, some involve farm animals (including horses), especially in animal assisted therapy settings (for overview, see Davis et al., 2015; Muñoz Lasa et al., 2015; Bert et al., 2016). Most AAI studies involving farm animals have been qualitative. The few randomized controlled trials conducted to date have indicated anxiolytic effects in humans (Berget et al., 2008, 2011; Pedersen et al., 2012). These studies have occurred in the context of Green Care, which uses ongoing farming activities on commercial farms in agricultural landscapes, including interactions with farm animals, as a basis for promoting human mental and physical health, social inclusion, and educational benefits (Working Group on the Health Benefits of Green Care, COST Action 866, 2010).

So far, no Green Care studies have reported on acute physiological changes in human participants when interacting with cows. Although farm animal species such as cattle are novel for urban human participants, their large size, big eyes, hair, warmth, and typically calm behavior may contribute to anxiolytic effects through associations with friendly familiar stimuli. The aim of this observational study was, therefore, to investigate the possible associations between interaction with well-socialized though unfamiliar cows and changes in female human peripheral OT levels and state anxiety. We predicted that, during a 15-min interaction, plasma OT would increase rapidly after commencing interaction with a cow whereas state anxiety would decline with increased time spent interacting with the cow. This prediction was based on previous findings in female dog owners, where plasma OT peaked 1 to 5 min after starting to interact with their dog, and cortisol was reduced 15 to 30 min after commencement of interaction (Handlin et al., 2011). With a predicted increase in OT and reduction in state anxiety over the course of the interaction, we expected to find a negative correlation between the OT and state anxiety responses to the interaction. In the absence of previous quantitative studies of this nature involving cows and given results showing benefits of interacting with companion animals, we tested our prediction in a standardized population of healthy participants in this initial exploratory study.

## 2. Materials and methods

### 2.1. Participants

Circulating OT levels tend to be higher in reproductively cycling women than in men (Bodo and Rissman, 2006), leading to the potential for stronger associations with social contexts in fertile women than in men and non-fertile women (MacDonald and MacDonald, 2010). Therefore, we recruited healthy female nursing students from Buskerud University College, Norway (*n* = 18), who were physically able to go to a farm and interact with cows in a cowshed. Recruitment was performed by one of us (GP), a nursing graduate, who visited different classes to explain the project. The exclusion criteria were pregnancy, breastfeeding, anxiety connected with blood sampling, fear of animals, allergies (apart from purely food allergies), psychiatric diagnoses or other severe illnesses, and medication (other than oral contraceptives). The participants were between 20 and 30 years of age, lived in a city and were unfamiliar with dairy farming although several had prior experience with horses. The number of participants was determined by (1) review of sample sizes used in published literature on human OT levels when interacting with companion animals (in the absence of comparable studies involving cows on farms), (2) availability of interested participants meeting the inclusion criteria, and (3) logistics of organizing the sampling sessions in an efficient manner that accommodated the students and the farmer.

### 2.2. Setting

The study was conducted on three consecutive Saturdays at a commercial organic farm with lactating Norwegian Red dairy cows that were well-socialized to humans. To avoid pseudo-replication, as well as repeated confinement in a pen separate from the herd, three cows participated in the intervention (one per day).

### 2.3. Ethical considerations

The study was conducted in accordance with the Declaration of Helsinki and approved by the Norwegian Regional Ethics Committee for Medical Research Ethics for studies involving humans (protocol 2010/1589). Because no experimental manipulations or painful procedures were performed on the cows, agricultural animal use did not require approval according to the Norwegian Regulations on Use of Animals in Research (Landbruks-og Matdepartementet, N, 2015). Before the study took place, participants were informed about the study procedures and signed a consent form. The farmer (owner of the animals) also provided informed consent. Samples and data were anonymized before storage and analysis. The farmer was present during the human-animal interactions to ensure safety of the participants and welfare of the cows. The farmer selected cows that he expected to be calm, gentle, and willing participants in interactions with unfamiliar people. The interactions were limited to 15 min of cow contact per participant to avoid participant fatigue and excessive grooming of cows. An agreement was made with the local medical clinic for treatment of any minor injuries, and general accident insurance was arranged for the duration of the study, but no negative incidents occurred. A confidentiality agreement was signed by the medical technician (phlebotomist) who collected blood samples.

### 2.4. Instruments

Level of anxiety was measured using the Norwegian version of the Spielberger State–Trait Anxiety Inventory-State Subscale (STAI-SS; Spielberger et al., 1970) as adapted by Haseth et al. (1990). The STAI-SS comprises 20 items. Participants indicate how they feel at the moment on a 4-point scale ranging from 1 – “almost never” to 4 – “almost always.” Ten items address the presence of anxiety symptoms, and the other 10 items concern the absence of anxiety symptoms. Items scoring the absence of anxiety symptoms were inverted before calculating the sum score (Spielberger, 1983). The total score, ranging from 20 to 80 points, reflects the level of state anxiety. Higher scores reflect greater levels of state anxiety than lower scores. Internal consistency was reported to have a median alpha coefficient of 0.94 (Spielberger, 1983).

### 2.5. Procedure

The human-animal interaction protocol was developed based on previous research performed with dogs and dog owners (Handlin et al., 2011), and involved a different cow on each study day. The cow was loose housed in a pen of approximately 10 m^2^ within the familiar environment of the cowshed, with food, water, bedding, and close visual contact with the other members of her herd. On sequential arrival at the farm, each participant was given protective overalls and safety boots to put on, and taken to a quiet waiting room to complete the STAI-SS. The participant was then left alone for 10 min with a neutral book to read before a qualified female phlebotomist entered to collect a first blood sample from an arm vein using a 21-gage needle (T1, baseline sample). Within 1–2 min of blood sampling, the participant was escorted into the cowshed by the farmer (male), who gave her a brush and briefly instructed her on how to interact safely with the cow and where to brush or stroke her. The instructions included speaking quietly, avoiding sudden movements, standing to the side of the cow and avoiding sensitive body regions. To ensure cow and participant safety, the farmer remained in the cowshed but stayed in the background, not talking with the participant during the intervention, while keeping an eye on proceedings and being ready to intervene if needed. No other people were present while the participant was interacting with the cow. No filming was undertaken in case it would impede recruitment or affect the participants’ responses during the interaction.

The intervention started when the participant entered the cow’s pen. After 5 min of interaction with the cow, including eye contact with the cow, speaking to her, and making body contact (e.g., with gentle brushes, strokes, scratches, and hugs), the phlebotomist entered the shed and asked the participant to step out of the pen and sit in a chair located 1.5–2 m from the cow, where a second blood sample was drawn (T2). Within 30 s of sample collection, the phlebotomist departed, and the participant re-entered the pen and continued interacting with the cow. After a further 10 min of cow interaction (total duration 15 min), the participant was guided back to the nearby waiting room where a final blood sample (T3) was collected, and the participant filled out a second STAI-SS (T3). On a separate sheet, the participant was asked “How did you experience being with the cow?,” with the response being selected from a 4-point Likert scale: 1 = very bad, 2 = bad, 3 = good, or 4 = very good. On the same sheet, there was an opportunity to describe the experience in her own words. When finished, the participant was checked by the phlebotomist to ensure no dizziness prior to departing from the farm.

### 2.6. Blood sampling

Due to the risk of contamination in the cowshed, intravenous catheterization for serial sampling was not permitted by the Norwegian Regional Ethics Committee for Medical Research. Therefore, we used standard venepuncture with a new needle for collection of each blood sample. For each collection, a maximum of two attempts were made. The blood samples were drawn into chilled 3 ml glass Vacutainer tubes containing K₃EDTA (1 mg/ml blood) and aprotinin (500 kIU/ml), and gently inverted 10 times. The tubes were centrifuged at 5,600 rpm for 5 min at 4°C and the plasma was pipetted into 1.75 ml Eppendorf tubes. The tubes were kept on dry ice for transport to the laboratory, where they were stored at −70°C until analyzed.

### 2.7. Assays

Oxytocin analysis was performed at Oslo University Hospital. Oxytocin levels were determined using Assay Designs’ Oxytocin Immunoassay (EIA) kit according to the manufacturer’s instructions (Enzo Life Sciences, Inc., Farmingdale, NY, United States; sensitivity range: 15.6–1,000 pg./ml; cross-reactivity with arginine vasotocin: 7.5%; intra-assay CV: <10% for values <80 pg./ml). Before assay, the samples were extracted using phenyl silica mini-columns (Bond Elute, Analytichem International, Harbor City, CA, United States) according to Sernia et al. (1991). During extraction, the sample concentration was doubled. The results were adjusted to account for this procedure. Recovery was checked by adding standards to the samples, which indicated close to 100% recovery.

### 2.8. Statistical analyses

Statistical analyses were performed using R software version 3.6.2. Oxytocin values at T1, T2 and T3 were compared in a repeated measures non-parametric Friedman test, and the STAI-SS scores at T1 and T3 were compared using a Wilcoxon’s matched pairs signed rank test. Pearson’s correlations were calculated to evaluate associations between change in OT levels (calculated as T2-T1, T3-T2, and T3-T1 values) and change in STAI-SS score (calculated as STAI SS at T3-T1). The level of significance was set at *p* < 0.05, and all tests were two-tailed.

## 3. Results

**Figure 1:**
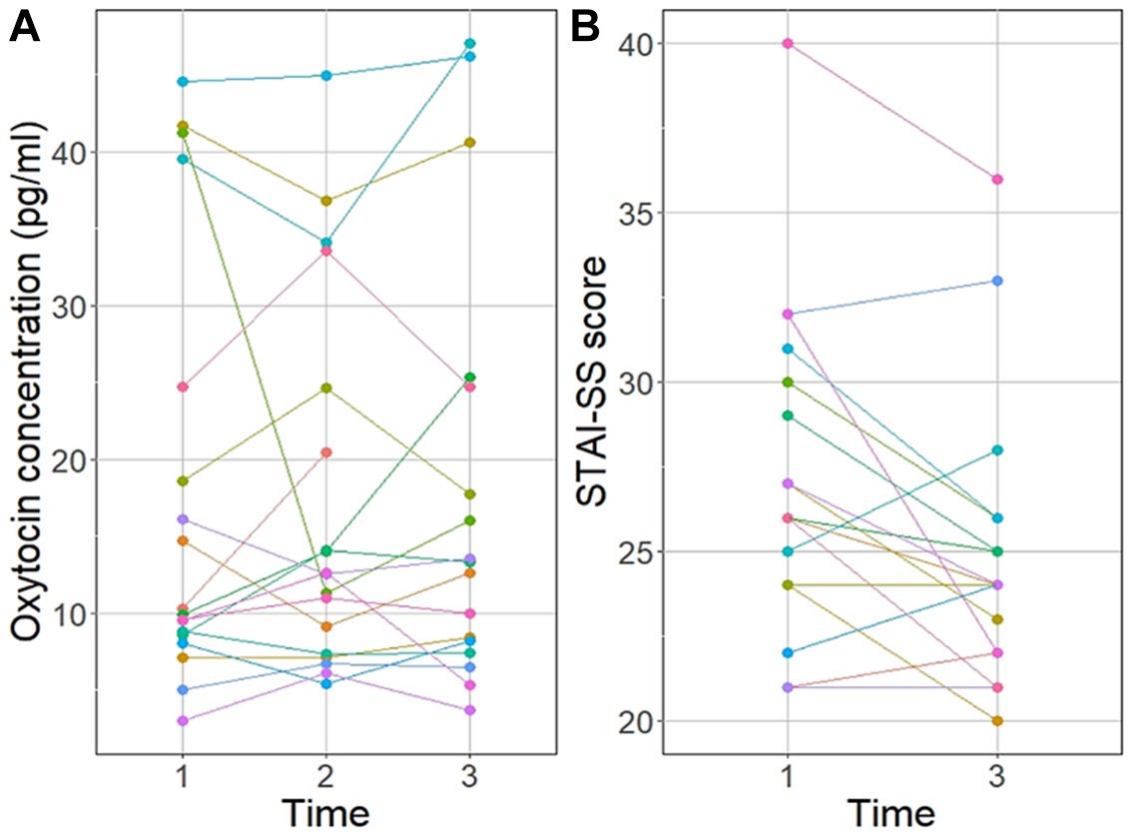
**(A)** Plasma oxytocin concentration (pg/ml) of 18 female nursing students at baseline (Time 1) and after 5 min (Time 2) and 15 min of interaction with a cow (Time 3), and **(B)** Spielberger State–Trait Anxiety Inventory-State Subscale (STAI-SS) scores at baseline (Time 1) and after 15 min of interaction with a cow (Time 3).

At the group level, plasma OT concentrations of participants at T1, T2 and T3 did not differ between time points (mean ± SD pg./ml, T1: 17.8 ± 14.1, range: 3.0–44.5; T2: 17.3 ± 12.2, range: 5.4–44.9; T3: 18.0 ± 14.1, range: 3.7–47.0; Friedman test: Chi^2^ = 0.64, *df* = 2, *p* = 0.728, Kendall’s W = 0.07; Figure 1A). Numerically, the OT level increased in 10 participants and declined in 7, both from T1 to T2 and from T2 to T3. Values fluctuated within individuals, with only 2 participants showing a consistent increase over time and none showing a consistent decline (Supplementary Table S1). Thirteen participants had higher OT at T2 or T3 compared to baseline (T1), but the difference was not significant at the group level (*p* < 0.05).

At the group level, mean state anxiety scores showed a small decline from TI (mean ± SD, 27.5 ± 4.8, range: 21–40) to T3 (25.4 ± 4.5, range, 20–36, Wilcoxon matched pairs signed rank test, V = 115.5, *p* = 0.015, *r* = 0.58, Supplementary Table S1). Numerically, 11 scores declined and 6 increased. All but one score fell within the low anxiety (20–39) range, with one T1 score indicative of moderate anxiety (40–59) and no scores indicating high anxiety (60–80) at either time point (Spielberger, 1983). The mean change in anxiety between T1 and T3 (calculated as T3–T1) was −2.1 ± 3.3 with a 95% CI of −0.49 – −3.73 (Figure 1B).

**Figure 2:**
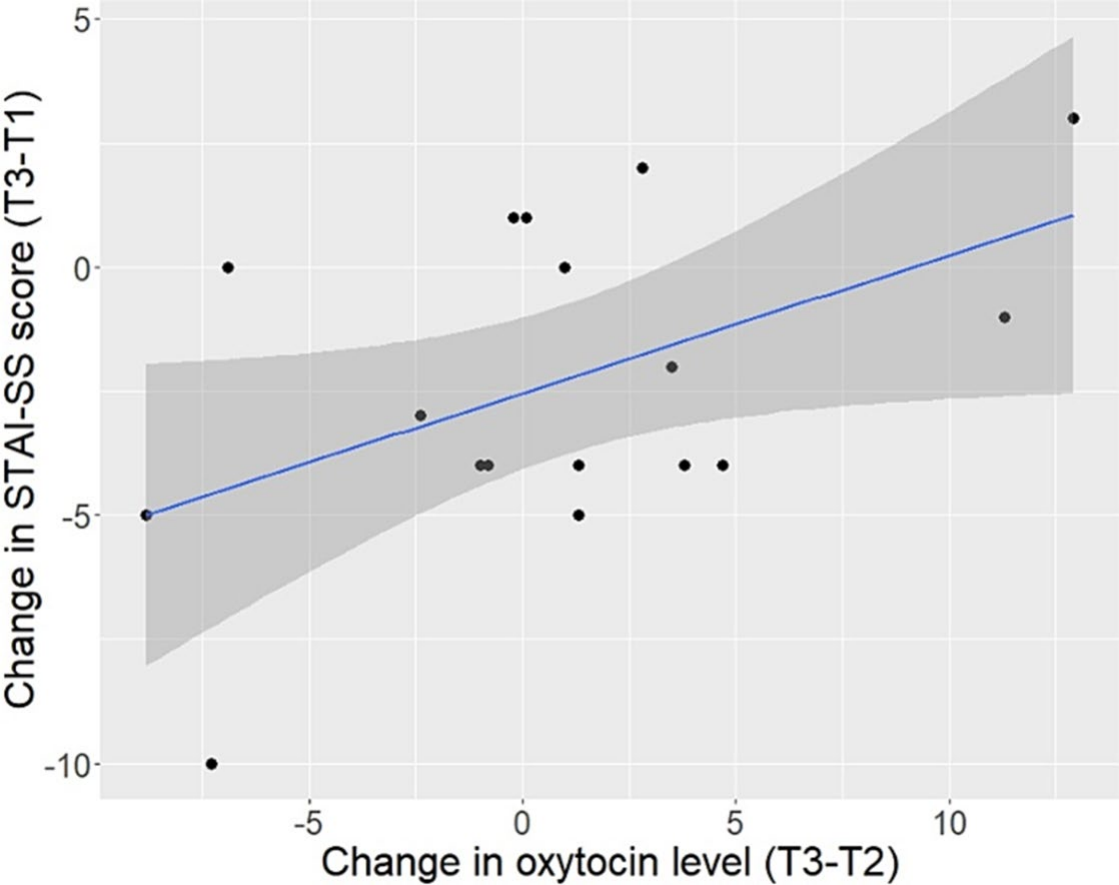
Relationship between the participants’ change in plasma oxytocin level (pg/ml) between 5 min (T2) and 15 min (T3) of interaction with a cow and change in the Spielberger State–Trait Anxiety Inventory-State Subscale (STAI-SS) score between baseline (T1) and (T3).

A significant positive correlation was found between the change in individual level of state anxiety between T1 and T3 and the change in OT concentration of the same individual between T2 and T3 (*r* = 0.49, *p* = 0.045; Figure 2). Five participants exhibited a drop in both STAI-SS score from T1 to T3 and OT concentration from T2 to T3 (although these correlated changes were minimal in all but 2 participants), while 2 responded with a corresponding increase in both STAI-SS values and OT. Without these individuals, this correlation disappears. Moreover, we detected no correlation between the change in anxiety scores from T1 to T3 and the change in OT from T1 to T2 (*r* < 0.01, *p* = 0.989) or T1 to T3 (*r* = 0.28, *p* = 0.282). Neither baseline (T1) STAI-SS score, nor difference in STAI-SS scores from T1 to T3, was related to baseline OT. The correlations between OT levels at different time points within the same subject were high (T1 and T2, *r* = 0.80, *p* < 0.001; T1 and T3, *r* = 0.86, *p* < 0.001; T2 and T3, *r* = 0.85, *p* < 0.001) and overall, the within-individual changes over time were relatively small.

Of the 18 participants, 16 (89%) responded that their experience with the cow was very good, and the remaining 2 described it as good (Supplementary Table S1). Qualitative responses included “Not scary, calm and cosy,” “Fun to be part of,” “The cow had a good effect on me,” and “Perhaps I’ll become a farmer instead.”

## 4. Discussion

Contrary to an expected rapid increase in OT after starting to interact with a cow, we found no significant groupwise change in plasma OT levels between the different time points (baseline, and after 5 and 15 min of interaction). This lack of group level differences between timepoints may be due to lack of a consistent time course of OT release across subjects, as reflected in the variation in findings between individuals. Given the short half-life of OT in blood (mean 3–7 min, Rydén and Sjöholm, 1969), it is not surprising that OT varied between individuals when sampled at set time points, as previously reported in healthy, non-pregnant and non-lactating women (Miller et al., 2009). More frequent sampling would be needed to detect the individual time course of episodic OT release into the circulation although this would require catheterization for serial blood sampling, which was not possible in the current study focused on responses in an on-farm setting. Our results show that interaction with the cows did not stimulate sustained release of OT into the circulation during either the first 5 min or the last 10 min of the intervention as a typical phenomenon across the participants. In general, the peripheral OT levels were low.

Several factors might explain inter-individual variation in plasma OT levels, despite high within-subject consistency across the 15-min intervention. One reason is variation in hormonal status across participants depending on their stage in the menstrual cycle and use of oral contraceptives (Ludwig and Leng, 2006; Farage et al., 2009). Peripheral OT levels are also influenced by the nature of social contact (Uvnäs-Moberg, 1998). Our choice of study population likely raised opportunities to detect socially mediated OT secretion given that the nursing profession attracts students with relatively high empathy characteristics (Wilson et al., 2012; Penprase et al., 2013). However, individual variation could have arisen from differences in the tactile characteristics and psychological quality of the interaction with the cow depending on the cow’s behavioral reactions during the interaction. For example, neck stretching when stroked and brushed appears to indicate pleasure in cows (Schmied et al., 2008), and Lürzel et al. (2020) found that its duration was positively correlated with cows’ peripheral OT levels whereas overall duration of human tactile contact showed no correlation. A perceived positive response from the cow could reinforce the rewarding quality of the interaction, potentially influencing the amount and timing of human OT release. Petersson et al. (2017) noted differences in human OT levels depending on the specific behavior occurring during interaction with a dog, with people who touched the dog more frequently having lower OT levels. Moreover, Geva et al. (2020) found that people who felt more able to communicate with the seal-like social robot, PARO, had lower OT levels than those who felt less able to communicate with PARO. Additionally, some participants had previous experience with horses, which may have increased their confidence around cows leading to inter-individual differences in OT response. Furthermore, variability in OT levels could have arisen due to differences in stress related to the venepuncture procedure (Deacon and Abramowitz, 2006), as acute stress has been reported to stimulate release of OT from the PVN into the peripheral circulation (in rats: Nishioka et al., 1998). While our recruitment of consenting nursing students limited this source of variability, there was likely some variation in transient anxiety associated with blood draws. Nevertheless, none of the participants withdrew from the study during data collection and all rated their experience with the cow as good or very good, indicating that, overall, the study conditions were perceived as positive rather than stressful.

Despite individual variation, some previous studies have shown an increase in peripheral OT in humans related to human-animal interaction over periods of 30 min or less (e.g., Odendaal and Meintjes, 2003; Miller et al., 2009; Handlin et al., 2011). The discrepancy between those findings and our results could be related to a difference in the nature of the relationship between the person and the animal, and the role of social attachment (Tops et al., 2007). For example, in an unfamiliar experimental setting, Handlin et al. (2011) detected elevated plasma OT in female dog owners (*n* = 10) after 1 or 3 min of physical interaction with their dog or 2 min afterwards compared to just before the interaction. Female (*n* = 10), though not male (*n* = 10), dog owners also had elevated circulating OT after 25 min of interaction with their dog starting when they returned home from work (Miller et al., 2009). In contrast, patients undergoing dialysis (3 women and 7 men) showed no change in circulating OT from before to immediately after a 1-h dog-assisted intervention on 11 intervention days with an initially unfamiliar dog (Menna et al., 2019). It is difficult to draw conclusions from comparison of results from studies involving animals to whom the participant is closely attached vs. unfamiliar animals given other differences (e.g., timing of sampling, sex ratio of participants). Nevertheless, in an fMRI study, a photo of a subject’s own dog was found to evoke brain activity similar to that observed when looking at photos of human family members whereas the image of an unknown dog did not lead to the same change in brain activity (Stoeckel et al., 2014). However, even among dog owners, interaction with their dog has produced varied OT results depending on the nature of the interaction (higher urinary OT associated with long eye contact, Nagasawa et al., 2009) and strength of attachment to the dog (rise in salivary OT associated with weak attachment, Powell et al., 2020). Marshall-Pescini et al. (2019) detected no change in the urinary OT of dog owners following interaction with their own dog vs. another familiar dog, and Curry et al. (2015) reported that the plasma OT response of dog owners to an unfamiliar dog was related to their prior dog ownership experience. Interestingly, Lürzel et al. (2020) detected no groupwise change in the peripheral OT concentration of cows following a 10-min grooming interaction with either a familiar researcher or an unfamiliar person. In contrast, D’Aniello et al. (2022) observed a positive correlation between cow serum OT and the duration of human-directed behavior (e.g., touch, push, lick, nibble) displayed toward the habitual caregiver but not an unfamiliar person during a 1-min “impossible task” scenario. Together, these results suggest that even a well-established individual caregiving relationship with the cow may not be consistently associated with socially mediated OT release in both humans and cows. In our study, the participants did not have an existing relationship with the cow. Furthermore, they were not familiar with interacting with any cows, precluding potential generalization from other cows to the cows in the current study.

Overall, the levels of state anxiety were low in our study, with only one participant having a marginally elevated basal STAI-SS score. Nevertheless, we detected a modest groupwise reduction in STAI-SS scores between T1 and T3, from the baseline level before the human-cow interaction to the post-interaction evaluation point. This change was detected with high effect size (*r* = 0.58) in our population of 18 female nursing students. A reduction in state anxiety (decrease in STAI-SS scores) of similar magnitude has been reported in college students (Wilson, 1991), including nursing students (Anderson and Brown, 2021), interacting with unfamiliar but friendly dogs. Following a three-month intervention with unfamiliar dairy cows and beef cattle for people with psychiatric disorders, Berget et al. (2011) found a reduction in the STAI-SS scores in the intervention group compared with the control group at a six-month follow-up. Pedersen et al. (2012) also noted a near significant reduction in the STAI-SS scores of clinically depressed patients between the start and end of a 12-week Green Care intervention on dairy farms as compared to treatment as is. Our findings indicate that a single, relatively short interaction with an unfamiliar cow resulted in a subjective perception of reduced anxiety in a majority of participants. It is possible that the novelty of the setting mildly elevated the basal STAI-SS scores, with habituation leading to a decline in scores by the end of the 15-min intervention. However, if a simple unconscious mechanism fully accounted for the change, then it might have been expected that the drop in scores would have been universal instead of occurring in 61% of the study population. All participants registered that the experience with the cow was positive, suggesting that the cow interaction, specifically, contributed to the lowered anxiety.

In our study, the change in an individual’s STAI-SS score between T1 and T3 was moderately correlated (*r* = 0.49) with the change in OT level of the same individual between T2 and T3, meaning that reduction in anxiety was associated with a drop in OT and vice-versa. This finding is contrary to our prediction that reduced anxiety would be associated with increased peripheral release of OT. That prediction was based on studies pointing to the involvement of OT in stress coping. For example, stimulating the intracerebral release of OT in the central amygdala resulted in a rapid attenuation of freezing in fear-conditioned rats (Knobloch et al., 2012), and peripheral OT release related to breastfeeding in healthy women was associated with increased stress resilience and lower anxiety (Cox et al., 2015). Handlin et al.’s (2011) study of owners interacting with their dogs shows that both OT and cortisol levels can rise and later fall in tandem over a period of 15 min. Nevertheless, because the parallel changes in OT levels and anxiety in our study were restricted to the tails of the population, we view the positive correlation between these variables with caution. While the changes in peripheral OT and anxiety levels of a majority of participants were not clearly related, this does not mean that anxiety was unaffected by OT. Friendly tactile contact during human-animal interaction may activate central oxytocinergic activity originating in parvocellular neurons of the PVN, lowering sympathetic activity and calming anxiety without concurrent release of OT into the circulation (Uvnäs-Moberg et al., 2015). Consistent with this hypothesis, Wredle et al. (2022) observed that gentle abdominal brushing during milking lowered cow plasma cortisol without elevating peripheral OT above that due to milking alone.

Regarding limitations and future research, this was an initial exploratory investigation of acute physiological changes and psychological perceptions associated with human interaction with cows. We detected a reduction in anxiety over time in close contact with cows, providing support for proceeding to randomized controlled trials to evaluate causal aspects of responses during interactions with cows, including the influence of having a well-established caregiving relationship with specific cows as opposed to interacting with unfamiliar cows (see Pedersen et al., 2011). Future studies should incorporate comparisons with farm visits excluding interaction with cows (positive control) and remaining at home (negative control). Only 40% of the measured OT levels fell within the sensitivity range of the OT assay, indicating that a larger sample size is needed in future work. While it was not feasible to catheterize people for serial blood sampling on the farm in our study, this might be possible at a research farm under more controlled conditions. Alternatively, repeated sampling of salivary OT could be used to capture individual differences in OT release on a broader scale, albeit that the OT origin, OT variant, and time course may differ relative to OT in blood (Uvnäs Moberg et al., 2020). Inclusion of measures of physiological stress such as serial blood cortisol levels would be useful for evaluating convergent validity with the detected changes in self-reported anxiety. In future studies, detailed behavioral measures would illuminate characteristics of interactions between people and cows (e.g., eye gaze, facial expressions, duration, and intensity of physical contact). The current study utilized a limited convenience sample from a single population of healthy young female nursing students and could be fruitfully expanded to include both females and males of additional populations including those with psychiatric disorders. There may also be benefits in therapies for children and the elderly (Mallon, 1994; Muñoz Lasa et al., 2015). We recommend gathering information about contraceptive use and stage in the menstrual cycle to account for these factors in future analyses on OT in women. In addition, assessment of blood progesterone and oestrogens would aid interpretation of results, or if blood sampling is not feasible, self-reported daily temperature recording might be used to establish cycle stage. We did not investigate trait anxiety in the current study due to the desire to avoid imposing a longer questionnaire on participants when requiring repeated responses over an interval of only 15 min. However, it could be useful to account for baseline trait anxiety in future studies. In addition, future studies should include additional farms to control for the possible influence of specific farms and farmers.

## 5. Conclusion

This is the first study to investigate associations between interacting with well-socialized farm animals, plasma OT levels and self-reported state anxiety in healthy people. Previous studies evaluating human OT release while interacting with companion animals vary considerably in participants, settings, interventions and outcomes (e.g., Miller et al., 2009; Handlin et al., 2011; Marshall-Pescini et al., 2019; Powell et al., 2020). The lack of a significant groupwise change in OT level in the current study illustrates the complexity of mechanisms involving OT as well as the complexity of technologies used to measure OT. The significant decline in state anxiety (STAI-SS) during a single 15-min interaction with a cow, and positive qualitative impression of the experience, is consistent with results from previous studies involving dogs (e.g., Wilson, 1991; Cole et al., 2007) and provides support for the inclusion of interactions with farm animals within the concept of Green Care.

## Data availability statement

The original contributions presented in the study are included in the article/Supplementary material. Further inquiries can be directed to the corresponding author.

## Ethics statement

The study was conducted in accordance with the Declaration of Helsinki and approved by the Norwegian Regional Ethics Committee for Medical Research Ethics for studies involving humans (protocol 2010/1589). Participants were informed about the study procedures and signed a consent form.

## Author contributions

BB, GP, and KU-M: conceptualization and methodology. GP: investigation and data curation. JV: formal analysis and visualization. BB, JV, GP, KU-M, and RN: interpretation, writing—original draft preparation and writing—review and editing. BB: supervision and project administration. All authors contributed to the article and approved the submitted version.

## Conflict of interest

The authors declare that the research was conducted in the absence of any commercial or financial relationships that could be construed as a potential conflict of interest.

## Publisher’s note

All claims expressed in this article are solely those of the authors and do not necessarily represent those of their affiliated organizations, or those of the publisher, the editors and the reviewers. Any product that may be evaluated in this article, or claim that may be made by its manufacturer, is not guaranteed or endorsed by the publisher.

## Supplementary material

The Supplementary material for this article can be found online at: https://www.frontiersin.org/articles/10.3389/fpsyg.2023.1252463/full#supplementary-material
