# Supplementary material for: Oxytocin levels and self-reported anxiety during interactions between humans and cows
Source: Front Psychol. 2023 Sep 14;14:1252463. doi: 10.3389/fpsyg.2023.1252463 (PMC10536144; doi:10.3389/fpsyg.2023.1252463)
Supplement: Supplementary file 1 [file Table_1.DOCX]

Supplementary Material

**Oxytocin levels and self-reported anxiety during interactions between humans and cows**

Bente Berget*, Judit Vas, Gunn Pedersen, Kerstin Uvnäs-Moberg and Ruth C. Newberry

*** Correspondence:** Bente Berget: bente.berget@usn.no

# Supplementary Data

Table S1. Plasma oxytocin concentration (OT) of 18 female nursing students at baseline (T1) and after 5 min (T2) and 15 min (T3) of interaction with a cow, Spielberger State-Trait Anxiety Inventory-State Subscale (STAI-SS) scores at T1 and T3 (where 20–80 represents the range of possible anxiety scores), and personal evaluation of the experience with the cow (where 1=very bad, 2=bad, 3=good, or 4=very good).

| **Participant** | **T1 OT (pg/ml)** | **T2 OT (pg/ml)** | **T3 OT (pg/ml)** | **T1 STAI-SS score** | **T3 STAI-SS score** | **Personal evaluation** |
| --- | --- | --- | --- | --- | --- | --- |
| 1 | 10.3 | 20.4 | missing | 21 | 22 | 4 |
| 2 | 14.7 | 9.1 | 12.6 | 26 | 24 | 4 |
| 3 | 7.1 | 7.1 | 8.4 | 24 | 20 | 4 |
| 4 | 41.7 | 36.8 | 40.6 | 27 | 23 | 4 |
| 5 | 18.6 | 24.6 | 17.7 | 24 | 24 | 4 |
| 6 | 41.2 | 11.3 | 16.0 | 30 | 26 | 3 |
| 7 | 9.9 | 14.0 | 25.3 | 26 | 25 | 4 |
| 8 | 8.6 | 14.1 | 13.3 | 29 | 25 | 3 |
| 9 | 8.8 | 7.3 | 7.4 | 32 | 33 | 4 |
| 10 | 39.5 | 34.1 | 47.0 | 25 | 28 | 4 |
| 11 | 44.5 | 44.9 | 46.2 | 31 | 26 | 4 |
| 12 | 8.0 | 5.4 | 8.2 | 22 | 24 | 4 |
| 13 | 5.0 | 6.7 | 6.5 | 32 | 33 | 4 |
| 14 | 16.1 | 12.5 | 13.5 | 21 | 21 | 4 |
| 15 | 3.0 | 6.1 | 3.7 | 27 | 24 | 4 |
| 16 | 9.5 | 12.6 | 5.3 | 32 | 22 | 4 |
| 17 | 9.6 | 11.0 | 10.0 | 40 | 36 | 4 |
| 18 | 24.7 | 33.5 | 24.7 | 26 | 21 | 4 |
| mean | 17.8 | 17.3 | 18.0 | 27.5 | 25.4 | 3.9 |
| SD | 14.1 | 11.2 | 14.1 | 4.8 | 4.3 | 0.3 |

**
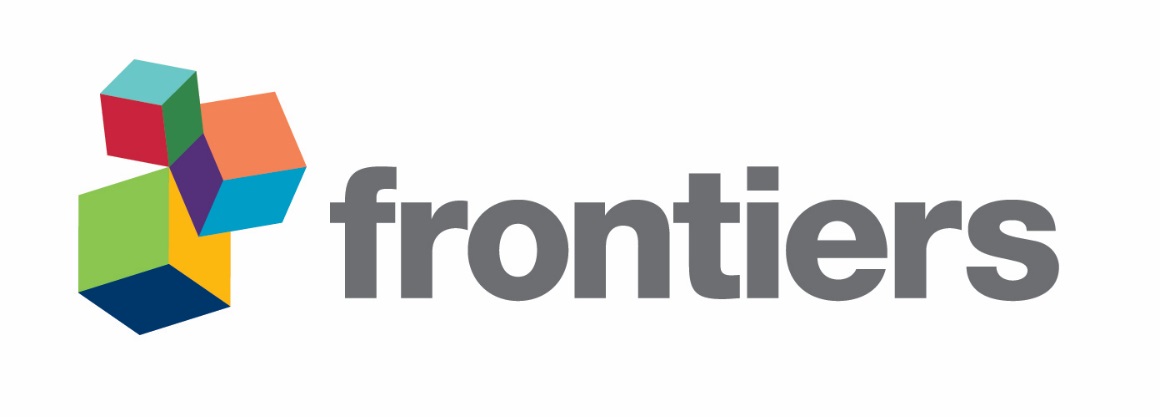
**
